# Bioengineered Silicon Diatoms: Adding Photonic Features to a Nanostructured Semiconductive Material for Biomolecular Sensing

**DOI:** 10.1186/s11671-016-1624-1

**Published:** 2016-09-15

**Authors:** Ilaria Rea, Monica Terracciano, Soundarrajan Chandrasekaran, Nicolas H. Voelcker, Principia Dardano, Nicola M. Martucci, Annalisa Lamberti, Luca De Stefano

**Affiliations:** 1 Institute for Microelectronics and Microsystems, Via P. Castellino 131, Naples, 80131 Italy; 2 Future Industries Institute, University of South Australia, Mawson Lakes Blvd, Adelaide, Australia; 3 Department of Biochemistry and Medical Biology, University of Naples “Federico II”, via Pansini 5, 80131, Naples, Italy

**Keywords:** Diatoms, Silicon, Photoluminescence, Biosensing

## Abstract

Native diatoms made of amorphous silica are first converted into silicon structures via magnesiothermic process, preserving the original shape: electron force microscopy analysis performed on silicon-converted diatoms demonstrates their semiconductor behavior. Wet surface chemical treatments are then performed in order to enhance the photoluminescence emission from the resulting silicon diatoms and, at the same time, to allow the immobilization of biological probes, namely proteins and antibodies, via silanization. We demonstrate that light emission from semiconductive silicon diatoms can be used for antibody-antigen recognition, endorsing this material as optoelectronic transducer.

## Background

Diatoms are unicellular organisms present in both fresh water and marine environments. The cell wall, called frustule, of diatom species is made of hydrogenated amorphous silica and shows a huge diversity in shape and size from the micro- to the nanoscale [1–3]. Hierarchical structure of diatom frustule is exactly reproduced by vegetative growth just exploiting the environment around. In recent years, diatom frustules, due to peculiar physical, chemical, and morphological properties, have been used in various biomedical, environmental, and technological applications as widespread available, three-dimensional, low-cost source of nanostructured silicon oxide-based rough material [4–8]. Even if size dimensions and pore architecture could fit lot of industrial technological applications, a definite limit in practical use of diatoms is due to the nature of their material: amorphous hydrogenated silicon oxide is scarcely conductive, so that it could not be used for any electrical application, including solar cells. Moreover, their surface is quite unreactive, and refractive index is lower or at least equal to that of rough glass. The range of potential applications for diatom frustules can be greatly enhanced by changing their material composition. It is well known by far that the natural silica of diatoms can be converted into silicon using a magnesiothermic reduction, preserving their shape and size [9–11]: gas/silica displacement reactions can convert biologically derived silica meso/nanostructures into new compositions, with retention of their fine features. These processes yield to new chemically tailored, complex-shaped, 3D meso/nanodevices for exciting technological applications: diatom frustules as photovoltaic material for dye-sensitized solar cells has been demonstrated using diatom silica coated with titanium dioxide [12]. Moreover, nanostructured diatom frustules from *Aulacoseira* sp. can be changed into nanostructured silicon semiconductors for solar energy conversion and power water electrolysis [11].

In this work, we describe the light emission properties of silicon-converted diatoms and investigate surface modification strategies with chemicals and biomolecules in order to realize an optoelectronic sensor for antibody-antigen recognition.

## Methods

### Chemicals and Materials

(3-Aminopropyl)triethoxysilane (APTES), bis(sulfosuccinimidyl)suberate (BS3), and H_2_SO_4_ were purchased from Sigma-Aldrich (MO, USA). Phosphate-buffered saline (PBS) was purchased from GIBCO (CA, USA). HCl was purchased from Romil (UK). Absolute ethanol and H_2_O_2_ were purchased from Carlo Erba (IT). Non-fat dried milk was purchased from EuroClone (IT). Protein A was purchased from Invitrogen (CA, USA). Mouse anti-His monoclonal antibody (AbaH) was purchased from Santa Cruz Biotechnology (CA, USA). Recombinant His-tagged p53 protein was kindly provided by Prof. Mariorosario Masullo.

Diatomite (*Aulacoseira* sp.) was a gift from Prof. Dusan Losic (University of Adelaide) and purified using the separation processes described elsewhere [13].

A simplified magnesiothermic reduction process, with respect to that firstly demonstrated by Sandhage group [9], was used to convert silicon dioxide of diatoms into pure silicon. We heated the magnesium (Mg) source and the diatom silica in a tungsten boat inside of a furnace to 650 °C under argon gas flow. After cooling, the process was repeated to ensure complete conversion. The Mg source and silica diatom frustules were thorough mixed without any other reducing agent in molar ratio of 1.25:1 Mg turnings to silica resulted in complete shape-preserving conversion. The reaction scheme is the following:

$$ 2\ \mathrm{M}{\mathrm{g}}_{\left(\mathrm{gas}\right)} + \mathrm{S}\mathrm{i}{{\mathrm{O}}_2}_{\left(\mathrm{solid}\right)} - > 2\mathrm{Mg}{\mathrm{O}}_{\left(\mathrm{gas}\right)} + \mathrm{S}{\mathrm{i}}_{\left(\mathrm{solid}\right)} $$

### Functionalization Procedure

The functionalization procedure of silicon-converted diatoms is based on silane chemistry [14]. Silicon frustules were first activated by Piranha solution (H_2_O_2_:H_2_SO_4_ 1:4) at 80 °C for 30 min, in order to create a surface rich of OH groups. Samples were extensively washed in milli-Q water to remove any adsorbed acid. Diatom frustules were then washed twice with deionized water and incubated in 5.0 M HCl solution overnight at 80 °C in order to remove metallic impurities. After HCl incubation, the diatom frustule dispersions were centrifuged for 30 min and the supernatant was removed. The pellet was washed twice with deionized water to remove excess of HCl.

Diatom dispersion was centrifuged for 30 min at 15,000 rpm and the supernatant was discarded. Structures were then silanized by immersion in 5 % APTES solutions in dry ethanol for 1 h at room temperature. Dry ethanol is used in order to avoid APTES hydrolysis in aqueous-based solution [15]. After this step, the sample was centrifuged for 30 min at 15,000 rpm and the supernatant was discarded. The functionalized diatoms were washed twice with absolute ethanol; the collected pellet was incubated for 10 min at 100 °C and then washed twice with ethanol and PBS (×1) buffer pH 7.4.

Protein A labelled with fluorescein isothiocyanate (FITC), in the following called PrA*, and not labelled, in the following called PrA, were immobilized on silane-modified diatoms using a bis(sulfosuccinimidyl)suberate (BS3) crosslinker. To this aim, each silicon diatoms sample (a pellet of few micrograms) was incubated with 1 mL of 1.6 mM BS3 in PBS solution (0.1 M; pH = 7.4) at 4 °C for 5 h. The functionalized sample was washed twice with PBS buffer and centrifuged for 30 min at 15,000 rpm. Each pellet was incubated overnight at 4 °C with 1 mL of 2 mg/mL PrA or PrA* in PBS (0.1 M; pH = 7.4) buffer. Protein A-conjugated silicon diatoms were incubated with 1.3 μM monoclonal antibody anti-His-tag in phosphate-buffered saline (PBS), pH 7.4, overnight at 4 °C. After two washes, the silicon diatoms were treated with 5 % non-fat dried milk in PBS at room temperature for 1 h to reduce non-specific peptide binding. After two washes, the sample was incubated with 100 μM recombinant His-tagged p53 protein in PBS ×1 buffer for 2 h at RT. The pellet was washed twice with PBS ×1 buffer to remove excess of His-tagged p53 protein.

### Scanning Electron Microscopy

The morphology of silicon-converted diatoms was investigated by scanning electron microscopy (SEM) using a field emission instrument (Zeiss-Supra 35). Diatoms dispersed in ethanol were deposited on a gold substrate. Images were acquired at 5-kV accelerating voltage and 30-μm wide aperture.

### Atomic and Electric Force Microscopy

Atomic-force microscopy (AFM) imaging of silicon diatoms was performed using a XE-100 AFM (Park Systems). Surface imaging was obtained in non-contact mode using silicon/aluminum-coated cantilevers (PPP-NCHR 10 M; Park Systems) 125-μm long with a resonance frequency of 200 to 400 kHz and nominal force constant of 42 N/m. Images, with a resolution of 256 × 256 pixels, were acquired with a set point of 15.8 nm and a sampling frequency of 0.5 Hz. Electric force microscopy (EFM) was performed at bias voltages of 0 V and 10 V.

### Fourier Transform Infrared Spectroscopy

Fourier transform infrared spectroscopy (FTIR) spectra were recorded by a Nicolet Continuμm XL (Thermo Scientific) microscope at 2 cm^−1^ resolution.

### Steady-State Photoluminescence

Steady-state photoluminescence (PL) spectra were excited by a continuous wave He-Cd laser at 325 nm (KIMMON Laser System). PL was collected at normal incidence to the surface of samples through a fiber, dispersed in a spectrometer (Princeton Instruments, SpectraPro 300i), and detected using a Peltier-cooled charge-coupled device (CCD) camera (PIXIS 100F). A long pass filter with a nominal cut-on wavelength of 350 nm was used to remove the laser line at monochromator inlet.

### Fluorescence Microscopy

Fluorescence microscopy images were acquired by means of a Leica MZ16 FA fluorescence stereomicroscope equipped with a Leica camera DFC320. The filter used for the acquisition was GFP2 consisting of a 460–40 nm band-pass excitation filter and a 510 nm barrier filter.

## Results and Discussion

**Fig. 1:**
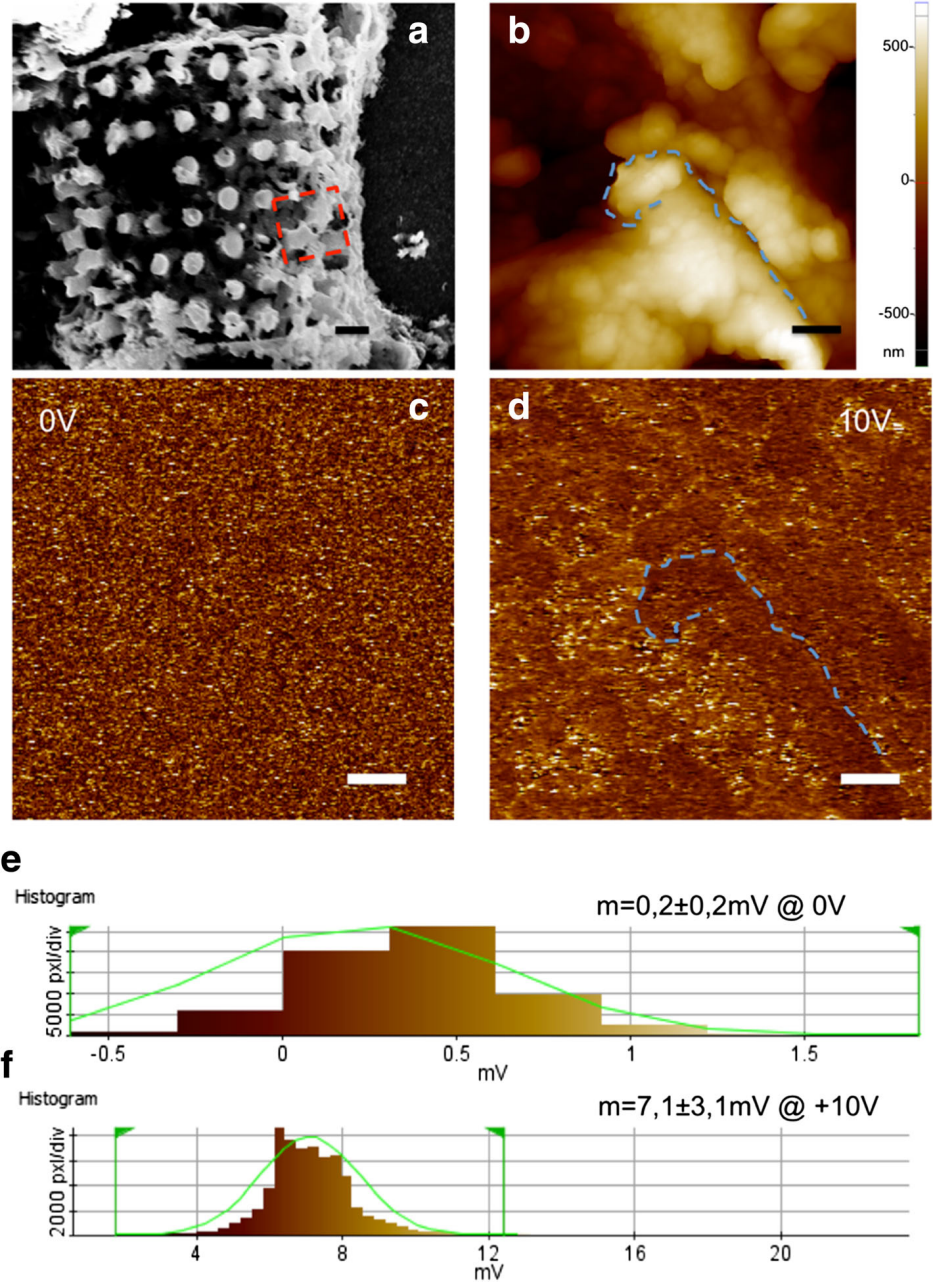
**a** SEM image of a silicon diatom (scale bar 3 μm): the *red square* underlines the region where AFM and EFM characterizations were performed. **b** AFM topography (scale bar 2 μm). EFM amplitude at tip bias voltage 0 V (**c**) and 10 V (**d**) (scale bar 2 μm): sample morphology appears only at 10 V bias voltage; EFM amplitude distribution at tip bias voltage 0 V (**e**) and 10 V (**f**): the Gaussian distribution shows that the average level rises when a voltage of 10 V is applied, indicating the conducting or semiconducting nature of the sample

Morphology of diatoms after the magnesiothermic reduction process was investigated by SEM and AFM analyses. SEM and AFM images, reported in Fig. 1a, b, show a porous structure with high-specific surface area and pores size of hundreds of nanometers: this peculiar morphology, preserved by reduction process as already demonstrated by Chandrasekaran et al. [10], makes silicon-converted diatoms (SiDs) an ideal material as transducer, assuring an efficient and rapid interaction with the species to detect [16–18]. Unlike the native diatoms made of silica, which is a good insulator material, SiDs are converted into a nanostructured semiconductor that widely opens the range of possible applications due to electrical features. A high-resolution electric characterization of silicon diatoms was performed by EFM; Fig. 1c, d shows EFM imaging of sample at bias voltages of 0 and 10 V, respectively. Any features of surface could not be identified at 0 V, while bright and dark zones following the porous nature of the sample were observed at 10 V. Moreover, the EFM amplitudes showed a Gaussian distribution (Fig. 1e, f) centered at a value that increased when a bias voltage of 10 V was applied. EFM analysis clearly demonstrated the semiconducting behavior of SiDs.

**Fig. 2:**
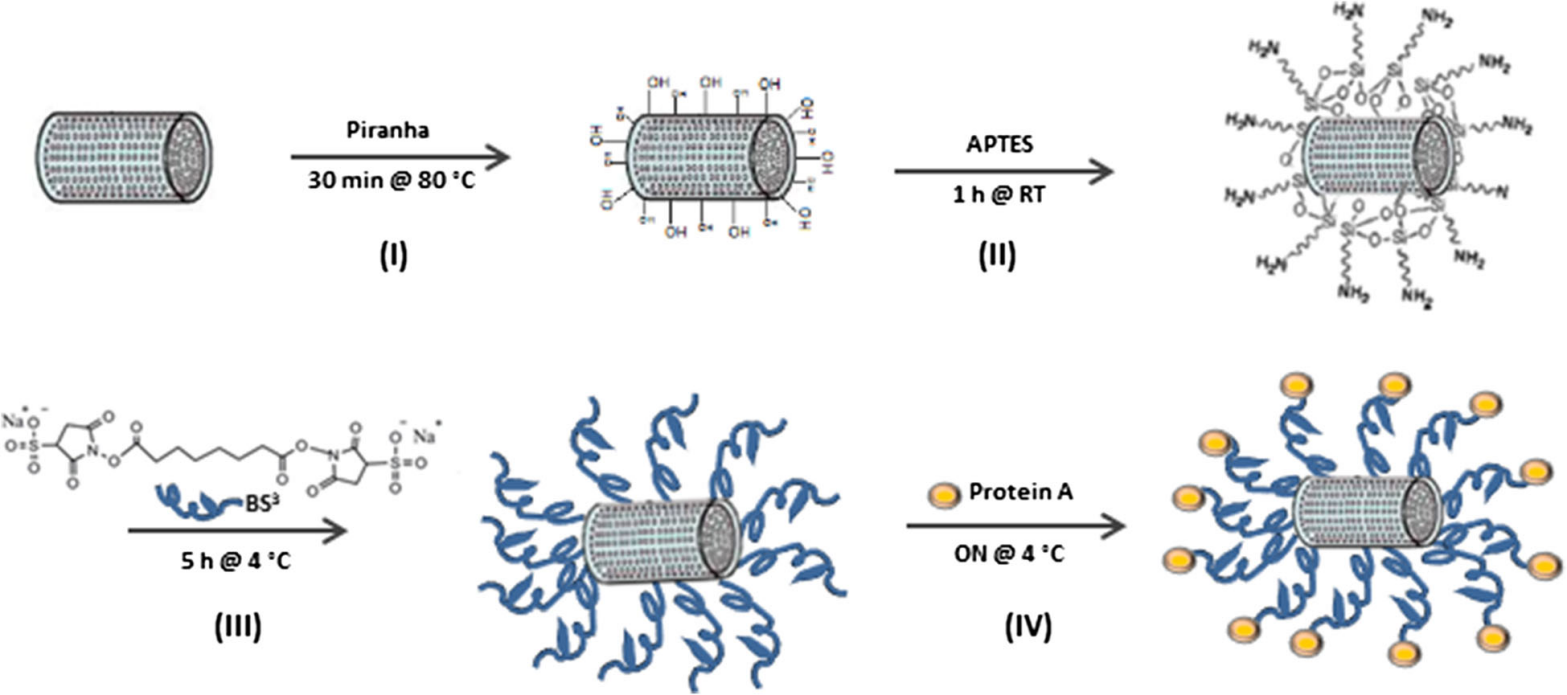
Scheme of functionalization procedure used for silicon diatoms

In a previous work, Chandrasekaran et al. demonstrated photocurrent generation from silicon diatoms converted via magnesiothermic process [10]. In this study, we showed that semiconductive SiDs could be also used as optical transducer for biomolecular recognition. To this aim, the surface of SiDs was chemically modified following the procedure schematized in Fig. 2. Briefly, Piranha treatment induced the formation of silanol groups (Si–OH) useful for APTES silanization (Fig. 2 I and II). Amine groups of APTES reacted with *N*-hydroxysulfosuccinimide (NHS) ester of BS3 forming a stable bond and releasing a NHS group (Fig. 2 III). The primary amines in the side chain of lysine residues of PrA reacted with the other NHS ester of BS3 forming an amine bond (Fig. 2 IV).

**Fig. 3:**
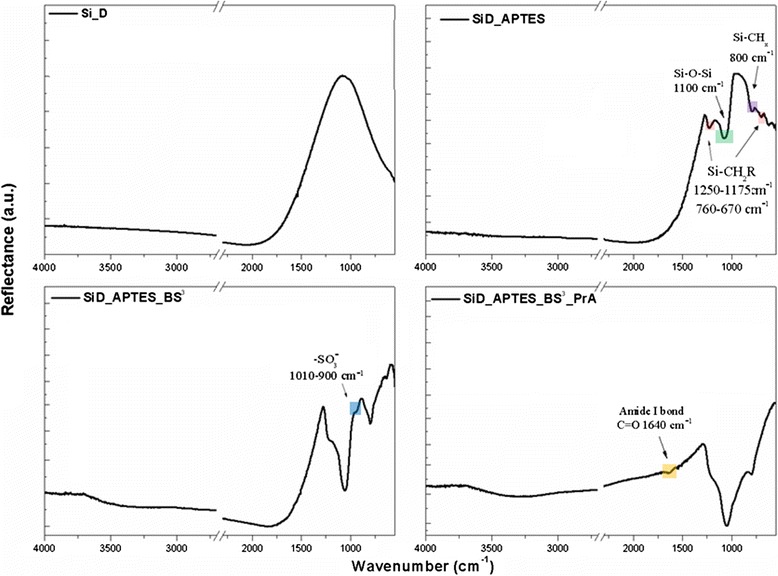
FTIR spectra of SiD after each functionalization step

Functionalization steps were monitored by FTIR spectroscopy: the infrared characterization is reported in Fig. 3. The FTIR spectrum of bare SiDs did not show any characteristic peak; after Piranha and APTES treatments, the peak related to Si–O–Si bond at 1100 cm^−1^ was well evident together with peaks of Si–CH_x_ (at 800 cm^−1^) and Si-CH_2_R (bands at 1250–1175 and 760–670 cm^−1^). BS3 functionalization was demonstrated by presence of a peak at about 950 cm^−1^ corresponding to sulphite ion. After PrA conjugation, a weak peak at 1640 cm^−1^ corresponding to C=O of peptide bond was observed. In order to better verify this conjugation step, the whole functionalization procedure (i.e., functionalization of silicon diatoms from Piranha to PrA) was repeated using FTIC-labelled PrA.

**Fig. 4:**
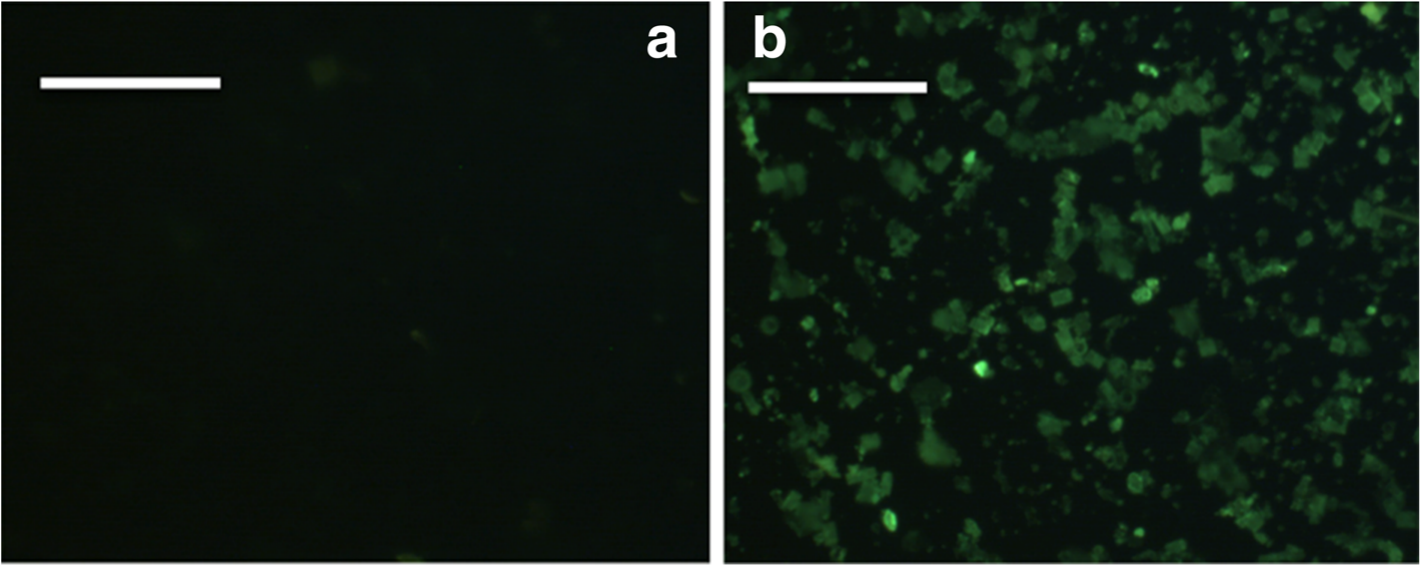
Fluorescence microscopy images of bare (**a**) and PrA* functionalized (**b**) SiD. Scale bar 100 μm

Fluorescence microscopy images of bare and PrA* functionalized SiD are shown in Fig. 4a green fluorescence (average intensity = 18 ± 2) was well evident in the case of sample functionalized with PrA*; conversely, bare sample appeared darker (average intensity = 10 ± 1). This result clearly demonstrated that the microshells of silicon diatoms were covered by fluorescent protein. We also investigated the fluorescence of diatoms after APTES and BS3 treatments in order to exclude a fluorescent contribution not coming from PrA*: no significant changes in the fluorescence of bare SiDs were observed (average intensity = 11 ± 2).

**Fig. 5:**
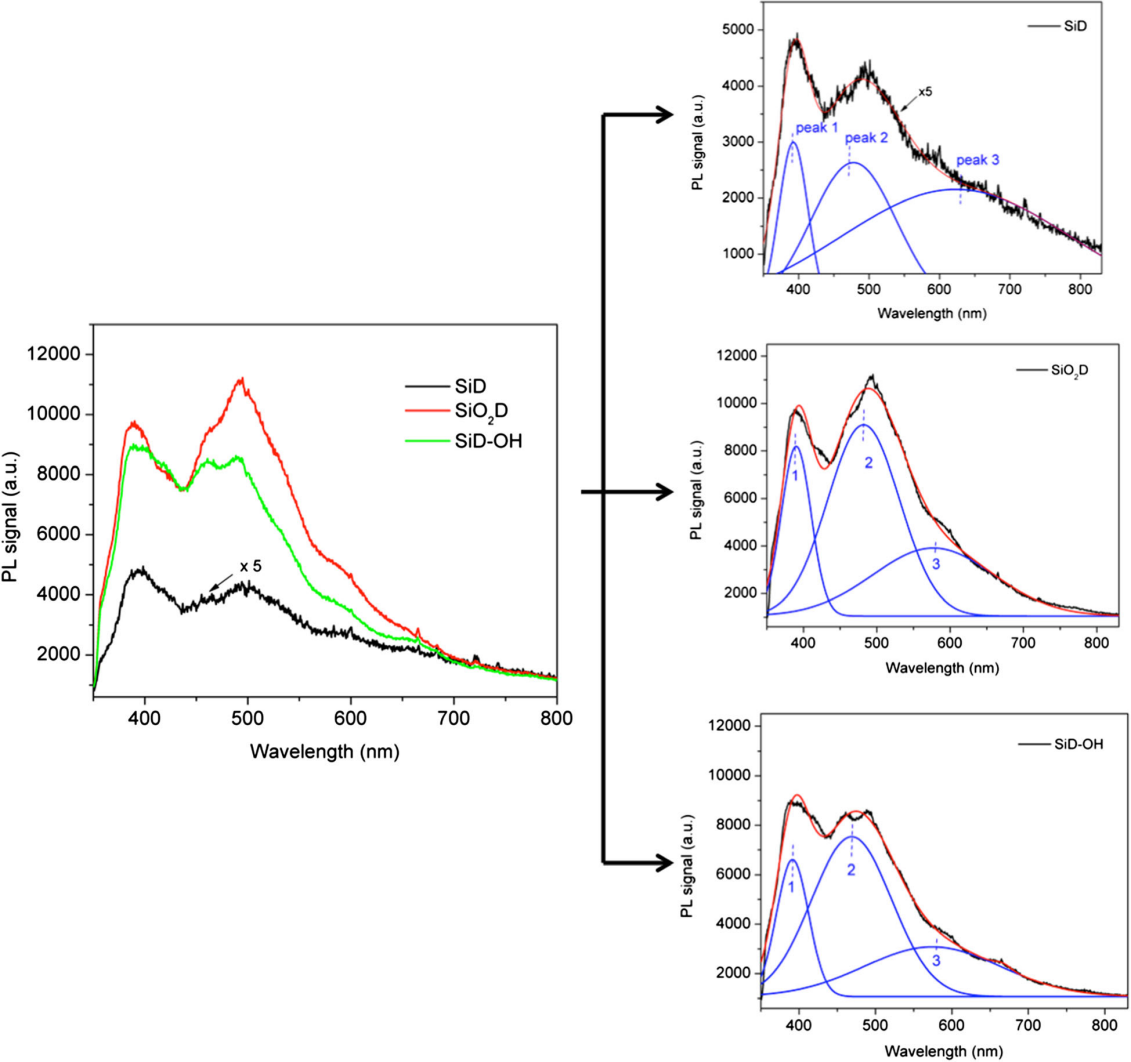
**a** Comparison between photoluminescence spectra of freshly converted silicon diatoms (SiD), silica diatoms (SiO_2_D), and silicon diatoms after incubation in water for 15 days (SiD-OH). **b** Spectra fitted by a Gaussian multi-peak routine

Further analysis was performed to study the silicon diatoms by steady-state photoluminescence. It is well known that under UV excitation (325 nm), fresh diatom frustules (SiO_2_D), made of amorphous silica, show a blue photoluminescence, clearly visible by naked eye. The origin of this light emission is not completely clear but it is commonly believed that it is mainly originated from surface defects including OH− groups and oxygen vacancies [19–21]: laser pumping generates excited electrons whose radiative decay produces photoluminescence emission. SiDs were characterized by a very weak photoluminescence signal due compared to SiO_2_D (Fig. 5a) and this was attributed to the removal of hydroxyl groups. The residual luminescence observed in SiD sample was due to a very low oxidation state of silicon surface. For SiDs stored in water for 15 days (SiDs-OH), the photoluminescence increased significantly, almost to the level of SiO_2_D.

Single photoluminescence spectra were fitted by Gaussian three peaks function (see the separated spectrum in Fig. 5b): in all cases, three main contributions to photoemission under laser excitation were observed. The first one, centered at 380–400 nm (peak 1), came from structural silanols (≡Si–OH), which were both inside and on the surface of the frustules; the intensity of this photoluminescence peak can vary depending on the size of the nanostructures where silanols are activated [18, 22]. The second peak at 480–500 nm (peak 2) could be ascribed to surface hydrogenated silicon groups (≡Si–H) [23]. A third weak peak, present at about 600–700 nm, was due to surface defects, such as oxygen vacancies [24], in agreement with PL emission recorded by Losic et al. in ref. [25], under laser excitation at 458 nm, resulting in emission at 682 nm; the other PL contributes that we registered (namely, peak 1 and 2) could not be excited at that wavelength.

**Fig. 6:**
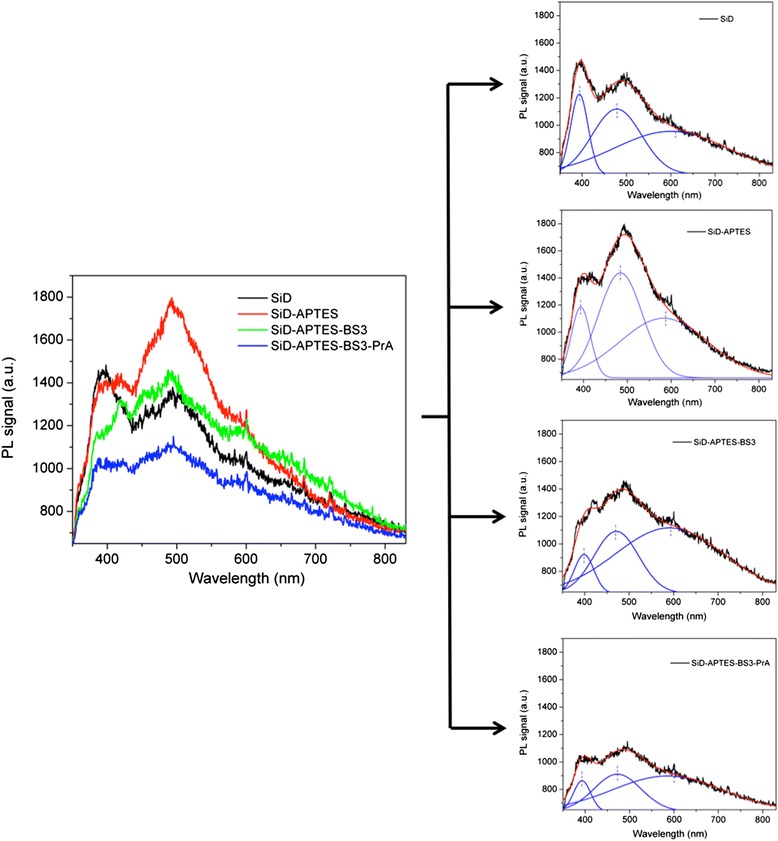
**a** Photoluminescence spectra of silicon diatoms after each functionalization step, from APTES to PrA. **b** Spectra fitted by a Gaussian multi-peak routine. **c** Ratio between the intensities of peak 2 and peak 1 for each functionalization step

Figure 6a shows a comparison between the photoluminescence spectra of silicon diatoms after each functionalization step, from APTES to PrA; photoluminescence spectra fitted by Gaussian three peaks function are also reported in Fig. 6b. These results highlighted that the bioconjugation procedure heavily affected photoluminescence emission from SiD frustules. The strong interaction between diatom surface and chemical/biological layers induced changes in the intensities of the main photoluminescence peaks. We found that the ratio between peak 2 and peak 1 intensities increased after each step and finally saturated, as summarized in Table 1; Fig. 6c reporting these values clearly shows the increasing trend. The chemical passivation and the bonding of PrA included a variety of non-covalent intermolecular phenomena such as hydrogen-bonding, electrostatic interactions, Van der Waals forces, and hydrophobic interactions which affected in a complex way the diatom surface and resulted in an excess of electrons available in the diatoms which could contribute to the overall photoluminescence increase: this is the well-known process of adsorption-assisted increase of exciton-phonon interaction [26].

**Table 1:** Ratio between the intensities of photoluminescence peaks 2 and 1 for each functionalization step

Functionalization step	Peak 2/peak 1 intensities ratio
SiD	2.35
SiD-APTES	3.46
SiD-APTES-BS3	3.78
SiD-APTES-BS3-PrA	3.80

**Fig. 7:**
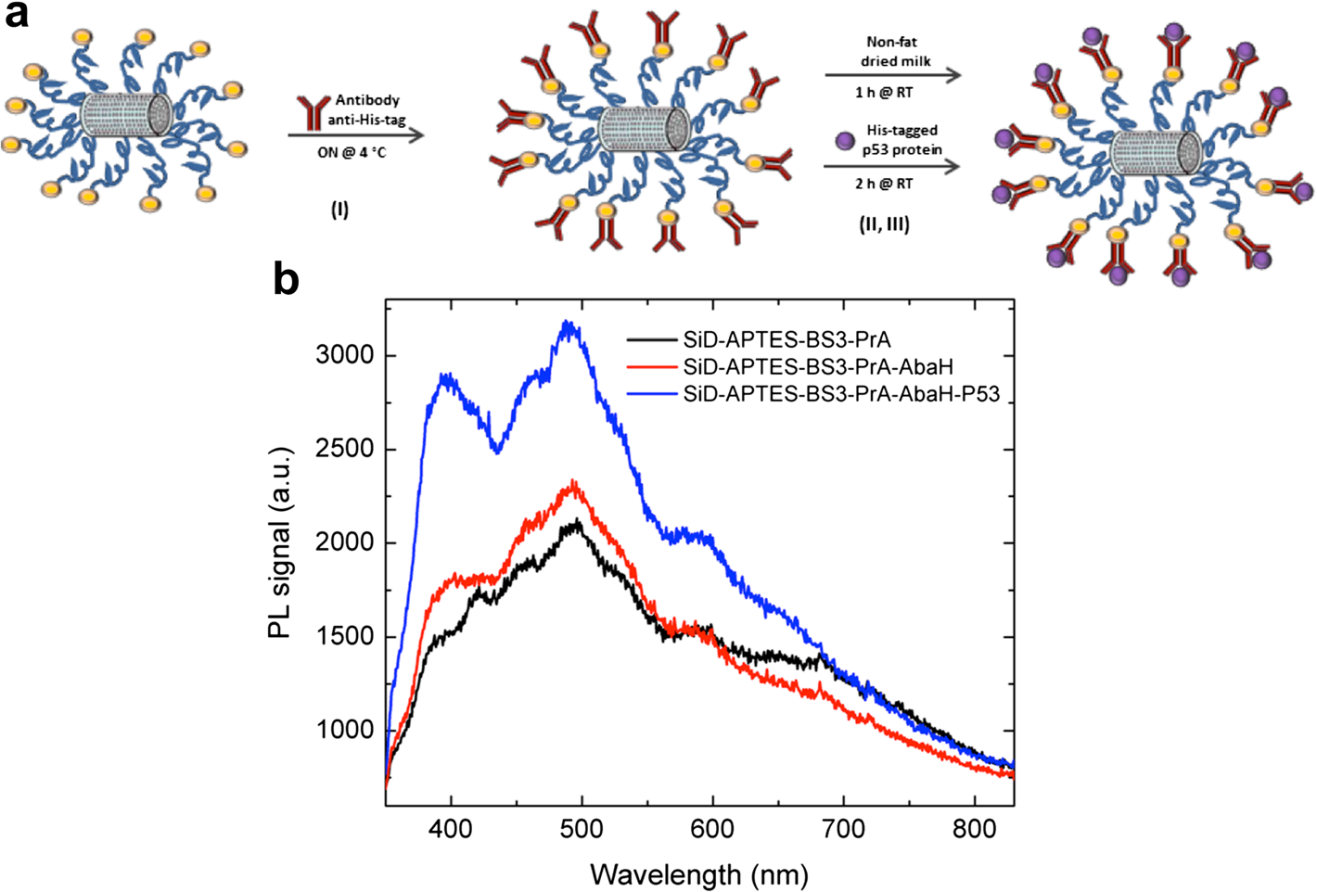
**a** Scheme of fuctionalization of silicon diatoms with anti-His-tag antibody (I) and incubation with His-tagged p53 protein (II). **b** Photoluminescence spectra of silicon diatoms after antibody-antigen interaction

A sample of SiD modified with PrA was conjugated to the mouse antibody anti-His-tag and then exposed to the His-tagged p53, a protein with an important role as tumor suppressor [27]. The scheme of functionalization process and antibody-antigen recognition is reported in Fig. 7a I and II, respectively. The interaction between the antibody anti-His-tag immobilized on silicon diatoms and its antigen, the His-tagged p53 protein, was monitored by a label-free optical technique based on the study of light emission from diatoms. After incubation of the antibody-functionalized frustules with p53 protein, a strong increase of the PL signal was detected, as it can be seen in Fig. 7b (blue curve).

## Conclusions

We demonstrated the light emission properties of semiconducting silicon-converted diatoms. The surface of silicon diatoms was chemically modified using a silane-based chemistry in order to attach a biological probe, the antibody anti-His-tag able to recognize the His-tagged p53 protein. The biomolecular interaction was demonstrated by a strong increase of the photoluminescence intensity emitted by silicon diatoms. These results endorse silicon diatoms as engineered biomaterial for optoelectronic sensing.
